# Delayed denervation-induced muscle atrophy in *Opg* knockout mice

**DOI:** 10.3389/fphys.2023.1127474

**Published:** 2023-02-22

**Authors:** Mingming Zhang, Ming Chen, Yi Li, Man Rao, Duanyang Wang, Zhongqi Wang, Licheng Zhang, Pengbin Yin, Peifu Tang

**Affiliations:** ^1^ Department of Orthopedics, Chinese PLA General Hospital, Beijing, China; ^2^ National Clinical Research Center for Orthopedics, Sports Medicine and Rehabilitation, Beijing, China; ^3^ Department of Orthopedics, The Second Affiliated Hospital of Harbin Medical University, Harbin, Heilongjiang, China

**Keywords:** denervation, muscle atrophy, OPG/RANKL/RANK, transcriptome sequencing, osteoporosis

## Abstract

Recent evidence has shown a crucial role for the osteoprotegerin/receptor activator of nuclear factor κ-B ligand/RANK (OPG/RANKL/RANK) signaling axis not only in bone but also in muscle tissue; however, there is still a lack of understanding of its effects on muscle atrophy. Here, we found that denervated *Opg* knockout mice displayed better functional recovery and delayed muscle atrophy, especially in a specific type IIB fiber. Moreover, OPG deficiency promoted milder activation of the ubiquitin-proteasome pathway, which further verified the protective role of *Opg* knockout in denervated muscle damage. Furthermore, transcriptome sequencing indicated that *Opg* knockout upregulated the expression of *Inpp5k*, *Rbm3*, and *Tet2* and downregulated that of *Deptor* in denervated muscle. *In vitro* experiments revealed that satellite cells derived from *Opg* knockout mice displayed a better differentiation ability than those acquired from wild-type littermates. Higher expression levels of *Tet2* were also observed in satellite cells derived from *Opg* knockout mice, which provided a possible mechanistic basis for the protective effects of *Opg* knockout on muscle atrophy. Taken together, our findings uncover the novel role of *Opg* in muscle atrophy process and extend the current understanding in the OPG/RANKL/RANK signaling axis.

## 1 Introduction

The osteoprotegerin/receptor activator of nuclear factor κ-B ligand/RANK (OPG/RANKL/RANK) signaling axis plays a crucial role in bone metabolism (Ono et al., 2020). Denosumab, a RANKL inhibitor, blocks RANKL binding to RANK, thus inhibiting osteoclast development and activity, decreasing bone resorption and increasing bone density (Kendler et al., 2021). Given its unique targets and mechanisms of action, denosumab is effective in the treatment of osteoporosis (Lyu et al., 2020). Non-etheless, given the crucial role of the OPG/RANKL/RANK axis in the immune response, some concerns remain regarding the side effects of this drug (Zhang et al., 2020). Previous studies have shown a potential adverse association with severe cellulitis and skin infections (Zhang et al., 2020), highlighting the need for further investigation of the effects of this axis on tissues other than bone to better characterize its biological function and identify potential side effects.

Considering the close relationship between bone and muscle tissues, the effects of the OPG/RANKL/RANK axis on the muscle have been studied (Dufresne et al., 2018; Hamoudi et al., 2019; Xiong et al., 2021). Results show that OPG-deficient mice develop muscle weakness and selective muscle atrophy (Hamoudi et al., 2020). RANKL inhibition and OPG supplementation improve muscle strength and restore bone mass in osteoporotic mice (Bonnet et al., 2019). Treatment with OPG-immunoglobulin fragment complex (OPG-Fc), RANK deletion, and RANKL inhibition alleviate muscle dystrophy in *mdx* transgenic mice (Dufresne et al., 2018, 2017; 2015). Moreover, OPG-Fc promotes muscle repair and regulates inflammation in cardiotoxin-induced acute muscle injury (Bouredji et al., 2021). In general, the effects of OPG/RANKL/RANK axis on muscle development, muscle injury, inherited muscle atrophy and age-related muscle atrophy have been preliminarily demonstrated. However, the role of this axis in secondary muscle atrophy requires further investigation.

Muscle atrophy occurs secondary to a variety of circumstances, including disuse, drug, malnutrition, cachexia and metabolic disease-induced muscle atrophy, in which disuse includes denervation, immobilization and hindlimb suspension (Xie et al., 2021). Muscle denervation is commonly observed in the clinic and is caused by trauma or is a symptom of neuromuscular disorders (Xie et al., 2021). Different from other secondary muscle atrophy models, denervation begins from the interruption of both efferent and afferent nerve fibers, in which gene expression profile change, protein homeostasis dysfunction, mitochondrial energy dysregulation, inflammation, oxidative stress, and autophagy are involved (Yang et al., 2020). However, no previous study has explored the role of the OPG/RANKL/RANK axis in this muscle condition.

In this study, we constructed a sciatic nerve transection model to induce muscle denervation in wild-type (WT) and *Opg* knockout mice. We report the first evaluation of the impact of the OPG/RANKL/RANK axis on denervation-induced muscle atrophy and present the possible mechanisms. These findings expand the current understanding of this crucial signaling axis and may provide basic evidence for the development of clinical treatment strategies.

## 2 Materials and methods

### 2.1 Animal experiments

All animal experiments were performed under a project license (No. 2021-X17-69), which was reviewed and approved by the Institutional Animal Care and Use Committee of Chinese PLA General Hospital. All experiments were performed in compliance with the national guidelines for the care and use of animal.

Homozygote *Opg* ^*−/−*^ (Tnfrsf11b^tm1Smoc^, NM-KO-00004) mice and WT mice were purchased from the Shanghai Research Center for Model Organisms. All mice were housed in specific-pathogen-free conditions under a 12/12-h light/dark cycle, and movement and feeding were not restricted. Mouse genotypes were determined *via* PCR amplification of DNA isolated from their tails. Twelve-week-old male WT and *Opg* ^*−/−*^ mice were used in our experiments to exclude the influence of hormones in female mice. A random allocation was performed for each genotype.

Sciatic nerve transection procedures were as follows: Mice were anesthetized using an intraperitoneal injection of 1% sodium pentobarbital. The sciatic nerve of the right hind limb was exposed and transected to a length of 10 mm (den group). The sciatic nerve of the left hind limb was exposed without transection (sham group). Body weight and gait of all mice were evaluated at the indicated time points. The mice were sacrificed before denervation, as well as days 3, 7, and 14 post denervation, and the bilateral gastrocnemius (GAS) muscles of all mice were harvested, weighed, and stored according to standard methods. The wet weight ratio was calculated by dividing the weight of the operative side by that of the sham-operated side.

### 2.2 Reagents

Information about reagents is listed in Supplementary Tables S1, S2.

### 2.3 Grip strength

The grip strength of mice was measured by allowing the mice to grip the metal mesh attached to the grip strength meter (Chatillon, US) and pulling them continuously. The test was repeated at least three times and the highest force for each test was recorded. The average grip strength of mice was calculated and normalized to body weight.

### 2.4 Footprint analysis

To evaluate changes in footprint and obtain gait images and parameters, CatWalk XT 9.0 (Noldus Information Technology, Wageningen, Netherlands) was used. Before and after denervation, mice were placed on a 1.3 m walkway and images of the footprints were captured using a high-speed camera. Three consecutive runs were conducted for each test. CatWalk XT software was used to analyze the footprint parameters, including stride length (length between successive placements of the same paw) and sway length (average width of the hind paws).

### 2.5 Hematoxylin and eosin (HE) staining and Masson’s trichrome staining

For HE staining, 10 μm-thick frozen GAS muscle slides were obtained and stained with HE according to the manufacturer’s instructions.

For Masson’s trichrome staining, GAS muscles were fixed with 4% paraformaldehyde (PFA), dehydrated and embedded in paraffin. 4 μm-thick paraffin-embedded GAS muscle slides were stained with Masson’s trichrome reagent according to the manufacturer’s instructions.

Histological slides were scanned using a PRECICE 500 (UNIC, China). ImageJ software (NIH, US) was used to quantify myofiber cross-sectional area (CSA) and minimal Feret diameter in HE slides, and fibrotic area in Masson slides. At least three random areas were analyzed for each slide.

### 2.6 Micro-computed tomography (CT) analysis

To analyze the bone mass and microarchitecture, micro-CT was performed using the Inveon MM system (Siemens, Munich, Germany), as previously described (Liu et al., 2019). Mouse femur samples were fixed in 4% PFA for 24 h and scanned. The following parameters were calculated using the Inveon Research Workplace (Siemens): bone mineral density (BMD, mg/cm^3^) and bone volume/total volume (BV/TV, %) in the region of interest on the femur (1-2 mm below the distal growth plate).

### 2.7 Cryosection staining

Fresh GAS muscles were mounted in optimal cutting temperature medium and snap-frozen in pre-cooled isopentane in liquid nitrogen. Frozen muscle sections (10 μm) were obtained and stored at −80°C. For fiber typing, the muscle sections were permeabilized and blocked with 0.3% Triton X-100 and QuickBlock blocking buffer at room temperature for 30 min. The sections were then incubated overnight at 4°C with the primary antibody solution which consisted of anti-Myhc I (BA-D5, DSHB), anti-Myhc IIa (SC-71, DSHB), anti-Myhc IIb (BF-F3, DSHB) and anti-dystrophin (ab15277, Abcam) diluted in QuickBlock primary antibody dilution buffer. After washing three times, the sections were incubated for 1 h at room temperature with a secondary antibody solution containing Alexa Fluor^®^ 405 (ab175652, Abcam), Alexa Fluor^®^ 488 (ab150121, Abcam), Alexa Fluor™ 568 (A-21124, Invitrogen) and Alexa Fluor^®^ 647 (ab172327, Abcam) diluted in QuickBlock secondary antibody dilution buffer. All sections were captured using confocal laser scanning microscopy (Leica TCS SP5, Germany), and the muscle CSA were analyzed using ImageJ software (NIH, US).

### 2.8 Western blotting analysis

GAS muscles were homogenized in RIPA lysis buffer supplemented with a protease inhibitor cocktail and the total protein content was estimated using a BCA protein concentration determination kit. Equal amounts of protein (20 μg) were separated *via* sodium dodecyl sulfate-polyacrylamide gel electrophoresis (SDS-PAGE), transferred to polyvinylidene difluoride membranes (PVDF; Bio-Rad) using a semi-dry transfer system (Bio-Rad) and blocked in EveryBlot Blocking Buffer. The membranes were incubated at 4°C overnight with the following primary antibodies: Anti-OPG, anti-MuRF-1, anti-Fbx32 and anti-GAPDH. The membranes were then incubated at room temperature with anti-rabbit or anti-mouse IgG-conjugated secondary antibodies for 1 h and visualized using Clarity Western ECL substrate. Band grayscale was analyzed using ImageJ software (NIH, US) and normalized to GAPDH.

### 2.9 RNA extraction and quantitative real-time PCR (qRT-PCR)

Total RNA was extracted from cell samples and GAS muscle samples using the FastPure^®^ Cell/Tissue Total RNA Isolation Kit V2 and cDNA was synthesized using the HiScript^®^ Ⅲ All-in-one RT SuperMix Perfect for qPCR. Subsequently, qRT-PCR was conducted in a CFX96™ thermal cycler (Bio-Rad, United States) using ChamQ Universal SYBR qPCR Master Mix according to the manufacturer’s instructions. The relative mRNA expression levels were calculated using the 2^−ΔΔCt^ method, and *Gapdh* was used as the internal control. Primer sequences used are listed in Supplementary Table S3.

### 2.10 Transcriptome sequencing and bioinformatics analysis

The transcriptomic data were deposited in the GEO database (GSE220152). Total RNA from GAS samples was extracted from the sham and den groups using the TRIzol reagent. The RNA-Sequencing library was constructed in a non-strand-specific manner and sequenced using Illumina Novaseq 6,000 by the Bestnovo Medical Laboratory (Beijing, China). Sequencing reads were mapped to the mouse genome reference build mm10 using STAR aligner. Transcripts per million and differentially expressed genes (DEGs) were calculated using RSEM and ebseq packages. DEGs were defined using the following criteria: FDR <0.05, and absolute log2 fold change >1. Gene ontology (GO) analysis was performed using the clusterProfiler package. Principal component analysis (PCA) plots, heatmaps, and volcano plots were visualized with ggplot2 and heatmap packages.

### 2.11 Fluorescence-activated cell sorting (FACS)

Satellite cells (SCs) were sorted as previously reported (Ryall et al., 2015). Briefly, hindlimb muscles from 6-week-old male mice were harvested, and blood, tendons, lipid tissues and nerves were removed. The muscles were finely chopped until a slurry was generated. The slurry was digested with 0.2% collagenase II and 0.1% dispase at 37°C for 60 min. The slurry was centrifuged, filtered and incubated with a primary antibody cocktail mixture as follows: APC-labeled α7-integrin, PE-labeled CD11b, CD31, CD45, and Sca1 and 7-AAD. Sorting was conducted on SH800S (SONY) and the gating strategy was based on positive α7-integrin, negative 7-AAD and CD11b/CD31/CD45/Sca1 staining.

### 2.12 Cell culture

The sorted SCs populations were cultured in collagen I-coated dishes. The culture medium was F10 basic medium supplemented with 20% fetal bovine serum, 1% penicillin-streptomycin and 10 ng/mL basic fibroblast growth factor. The growth medium was changed every other day. The proliferation ability of SCs was evaluated using the BeyoClick EdU Cell Proliferation Kit following the manufacturer’s instructions. Briefly, EdU was added into the culture medium and incubated for 6 h. The cells were fixed and permeabilized and click additive solution and Hoechst 33,342 were added. The proliferation ability of SCs was calculated by dividing the number of EdU-positive cells by the total number of cells. To induce myogenic differentiation, 20,000 SCs/well were seeded in a 48-well plate coated with collagen I and cultured in a differentiation medium (2% horse serum and 1% penicillin-streptomycin in DMEM) for 48 h. The differentiation ability of SCs was evaluated using myotube staining. Briefly, myotubes were fixed with 4% PFA, permeabilized and blocked with 0.2% Triton X-100 and QuickBlock blocking buffer at room temperature, respectively. The myotubes were incubated with a primary antibody against MyHC (MF20) overnight at 4°C and subsequently incubated with the secondary antibody goat anti-mouse Alexa Fluor 568 at room temperature. The cells were counterstained with DAPI. The differentiation index was calculated by dividing the number of nuclei per myotube by the number of DAPI^+^ nuclei (all MyHC^+^ myotubes were considered). The fusion index was calculated by dividing the number of nuclei per myotube by the number of DAPI^+^ nuclei (only MyHC^+^ myotubes with two or more nuclei were considered).

### 2.13 Statistics

Statistical analysis was conducted using GraphPad Prism 8.0 (GraphPad) and data were presented as the mean ± standard deviation (SD). Comparisons between multiple groups were evaluated using one-way ANOVA followed by Tukey’s test. A two-tailed unpaired Student’s t-test was used to compare differences between two groups. *p* < 0.05 was considered statistically significant.

## 3 Results

### 3.1 *Opg* knockout mice displayed the similar functional recovery with WT mice after denervation

**FIGURE 1:**
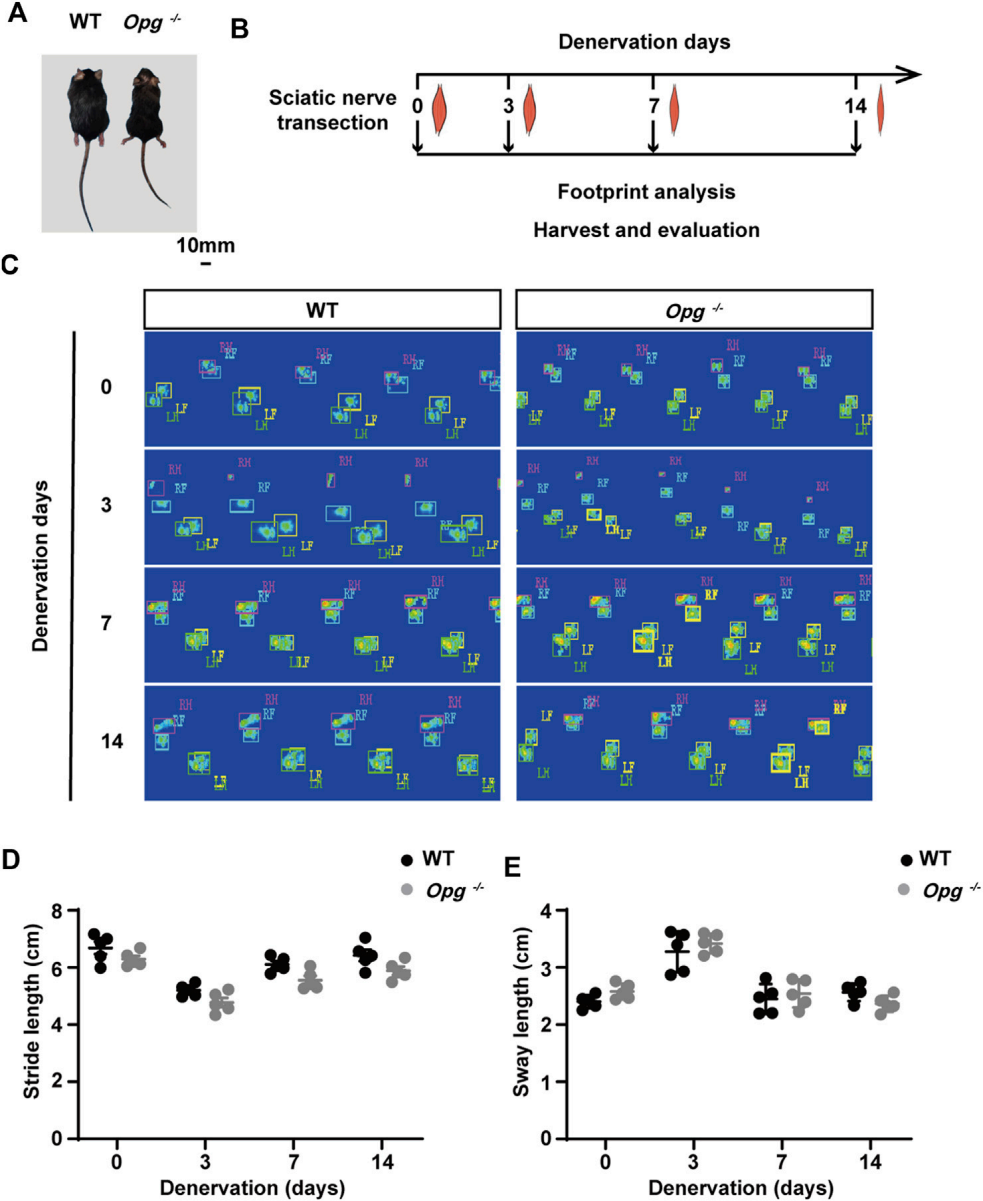
*Opg* knockout mice exhibited the similar functional recovery with WT mice after denervation. **(A)** Body size comparison of 12-week-old WT and *Opg* ^*−/−*^ mice. Scale bar 10 mm. **(B)** Schematic of the experimental design to evaluate muscle atrophy at indicated time points. **(C)** The representative footprints of WT and *Opg* ^*−/−*^ mice at indicated time points. **(D-E)** Quantitative analysis of footprints to evaluate differences in stride length **(D)** and sway length **(E)**. *n* = 5/group. Data are represented as the mean ± SD.

Before we explored the effects of the *Opg* knockout on denervated muscle atrophy, we first verified the known phenotypes of the *Opg* ^*−/−*^ mice we established. Compared to WT mice, reduced body mass was observed in 3-month-old *Opg* ^*−/−*^ mice (Figure 1A and Supplementary Figure S1B). Further, 3-month-old *Opg* ^*−/−*^ mice developed an osteoporotic phenotype, as indicated by lower BMD (Supplementary Figure S1C) and BV/TV (Supplementary Figure S1D) compared to WT mice. Meanwhile, normalized to body weight, the weight of the GAS (mixed-type muscle) and whole limb grip strength were comparable in 3-month-old *Opg* ^*−/−*^ mice and age-matching WT mice (Supplementary Figures S1E, S1F), which was in line with what was reported previously (Hamoudi et al., 2020).

Next, sciatic nerve transection models were established in WT and *Opg* ^*−/−*^ mice and footprint analysis was performed before denervation and on days 3, 7 and 14 post denervation to evaluate the effects of nerve transection on WT and *Opg* ^*−/−*^ mice (Figure 1B). Representative footprints from the sham and injury conditions were shown (Figure 1C). As motor function was affected by both nerve and muscle, we compared the motor function by using static paw parameters, stride length and sway length (Li et al., 2021). We found that motor function was significantly impaired on day 3 post denervation and gradually recovered on days 7 and 14, which seemed no significant difference between WT and *Opg* ^*−/−*^ mice (Figures 1D, E). Collectively, the above results indicated that denervation severely impaired nerve and muscle function and *Opg* ^*−/−*^ mice displayed the similar functional recovery with WT mice after denervation.

### 3.2 *Opg* knockout delayed GAS muscle atrophy after denervation

**FIGURE 2:**
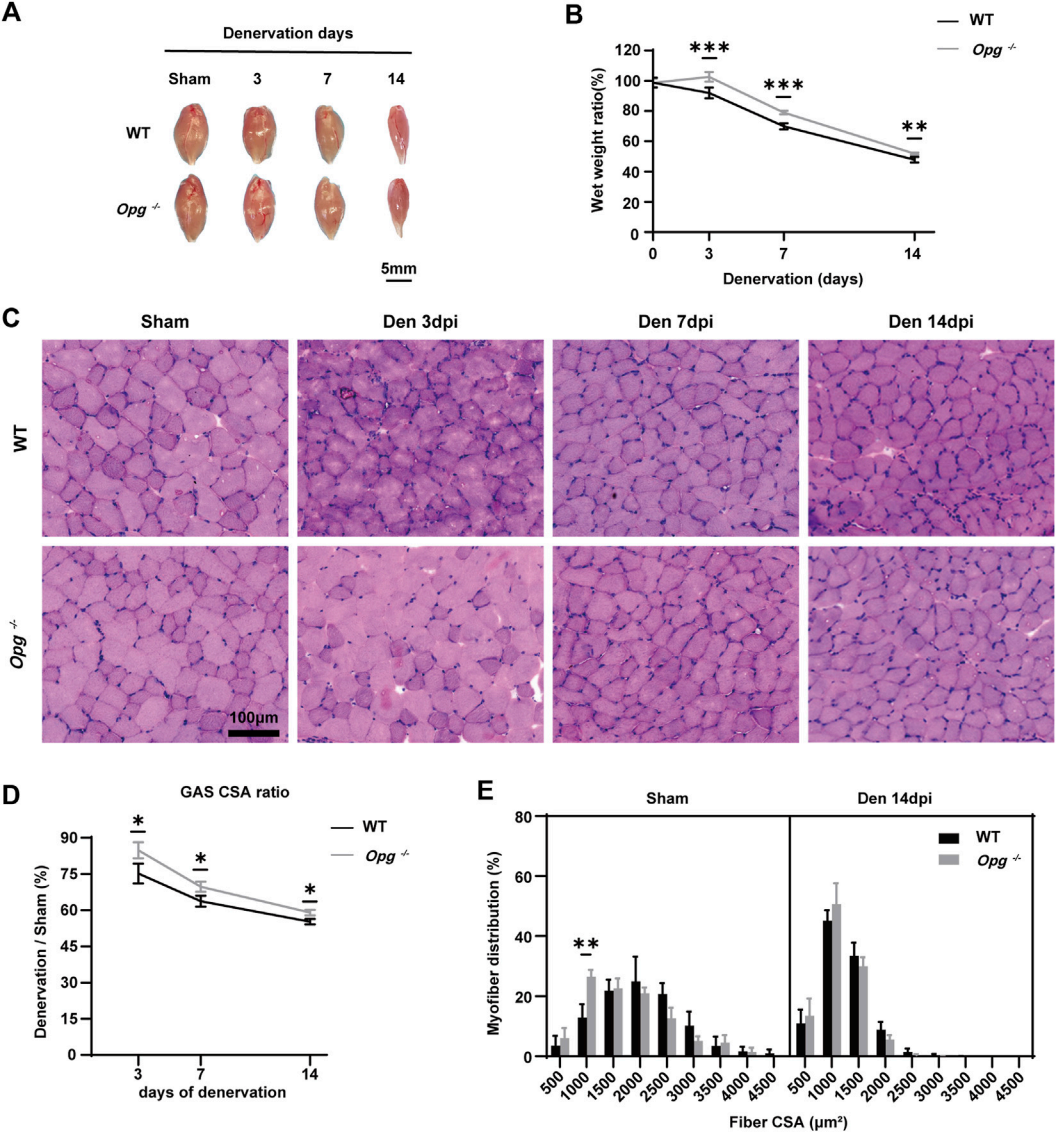
*Opg* knockout delayed the GAS muscle atrophy after denervation. **(A)** Gross morphology of gastrocnemius muscles, harvested from WT and *Opg* ^*−/−*^ mice days 3, 7 and 14 after denervation. Scale bar 5 mm. **(B)** The wet weight ratio of gastrocnemius muscles at the indicated time points after denervation (*n* = 5/group). **(C)** HE staining of cross-sections of gastrocnemius muscles to analyze CSA at the indicated time points after denervation. Scale bar 100 μm. **(D)** The CSA ratio of gastrocnemius muscles at the indicated time points after denervation (*n* = 3/group). **(E)** Myofiber CSA distribution of gastrocnemius muscles from WT and *Opg* ^*−/−*^ mice 14 days after denervation. *n* = 3/group. Data are represented as the mean ± SD. ∗*p* < 0.05, ∗∗*p* < 0.01, ∗∗∗*p* < 0.001 compared with WT mice.

To evaluate muscle loss, GAS muscles of each mouse were harvested before denervation and on days 3, 7 and 14 after denervation (Figure 2A) and the wet weight ratio was calculated. During the first 2 weeks of denervation, the wet weight ratio of GAS muscle decreased rapidly in both WT and *Opg* ^*−/−*^ mice, whereas the wet weight ratio of *Opg* ^*−/−*^ mice was higher than that of WT mice on days 3, 7 and 14 after denervation, which suggested that *Opg* ^*−/−*^ mice showed delayed GAS muscle atrophy (Figure 2B). HE staining was performed to further evaluate the effects of denervation on muscle pathology (Figure 2C). Firstly, there are increased nuclear volume, heteromorphic nuclei, vacuolated nuclei and increased number of nuclei in the course of denervation in WT and *Opg* ^*−/−*^ mice. Moreover, we evaluated the muscle fiber CSA of WT and *Opg* ^*−/−*^ mice. Regardless of denervation, the CSA and mean minimal Feret diameter of *Opg* ^*−/−*^ mice were significantly lower than those of the WT mice, suggesting a muscle atrophy phenotype in *Opg* ^*−/−*^ mice, which was consistent with previous report (Hamoudi et al., 2020) (Supplementary Figures S2A, S2B). After denervation, the GAS CSA ratio was significantly reduced in both WT and *Opg* ^*−/−*^ mice, but the ratio in *Opg* ^*−/−*^ mice was significantly higher than that in WT mice, demonstrating a delayed muscle atrophy (Figure 2D). Myofiber distribution analysis showed that the peak of CSA distribution in WT mice was higher than that in *Opg* ^*−/−*^ mice before denervation and *Opg* ^*−/−*^ mice had a higher percentage of small myofibers (<1,500 μm^2^) (Figure 2E). Denervation reduced myofiber CSA and the CSA distribution was not significantly different between the WT and *Opg* ^*−/−*^ mice (Figure 2E), which further supported the delayed muscle atrophy in *Opg* ^*−/−*^ mice. Denervation induced muscle fibrosis 14 days after nerve transection, as indicated by Masson’s trichrome staining (Supplementary Figure S2C). However, the fibrotic area observed in *Opg* ^*−/−*^ mice was significantly lower than that in WT mice (Supplementary Figure S2D). In general, GAS muscle atrophied rapidly upon denervation and *Opg* knockout delayed muscle atrophy and alleviated fibrosis caused by denervation.

### 3.3 *Opg* knockout mice showed a milder activation of the ubiquitin-proteasome pathway after denervation compared to WT mice

**FIGURE 3:**
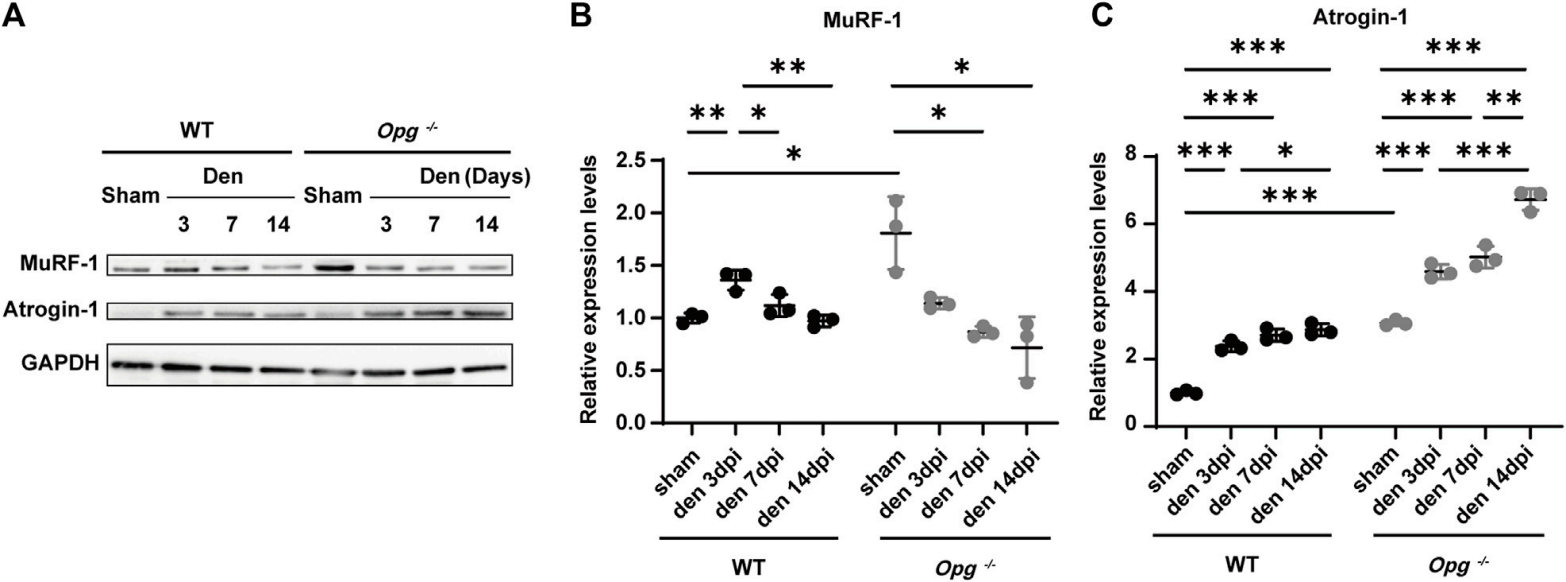
*Opg* knockout delayed the GAS muscle atrophy *via* the ubiquitin-proteasome pathway. **(A–C)** The expressions of E3 ubiquitin ligases MuRF-1 and Atrogin-1 quantified *via* western blotting in gastrocnemius muscles at indicated time points after denervation. *n* = 3/group. Data were represented as the mean ± SD. ∗*p* < 0.05, ∗∗*p* < 0.01, ∗∗∗*p* < 0.001.

Ubiquitin-proteasome system is an important proteolytic system with a significant role in muscle atrophy (Parveen et al., 2021). To further explore the effects of *Opg* knockout on denervated muscle, we measured the expression of E3 ubiquitin ligases MuRF-1 and Atrogin-1 in GAS muscles. The results showed the higher expression of MuRF-1 and Atrogin-1 in *Opg* ^*−/−*^ sham group than WT sham group (Figures 3A–C), suggesting the undergoing muscle atrophy in *Opg* ^*−/−*^ mice, which was the same as previous reports (Hamoudi et al., 2020). After denervation, compared to respective sham group, the expression of MuRF-1 and Atrogin-1 was upregulated in WT mice, whereas that in *Opg* ^*−/−*^ mice was discrepant (Figures 3A–C). MuRF-1 was downregulated and Atrogin-1 was upregulated in *Opg* ^*−/−*^ mice, whereas the increase fold of Atrogin-1 in *Opg* ^*−/−*^ mice was lower than that in WT mice after denervation relative to sham group (Figures 3A–C). These results suggested that the ubiquitin-proteasome pathway was activated upon denervation and *Opg* knockout mice exhibited milder activation compared to WT mice, which further supported the protective role of *Opg* knockout.

### 3.4 Denervation promoted muscle fiber type transition and *Opg* knockout delayed type ⅡB myofiber atrophy after denervation

**FIGURE 4:**
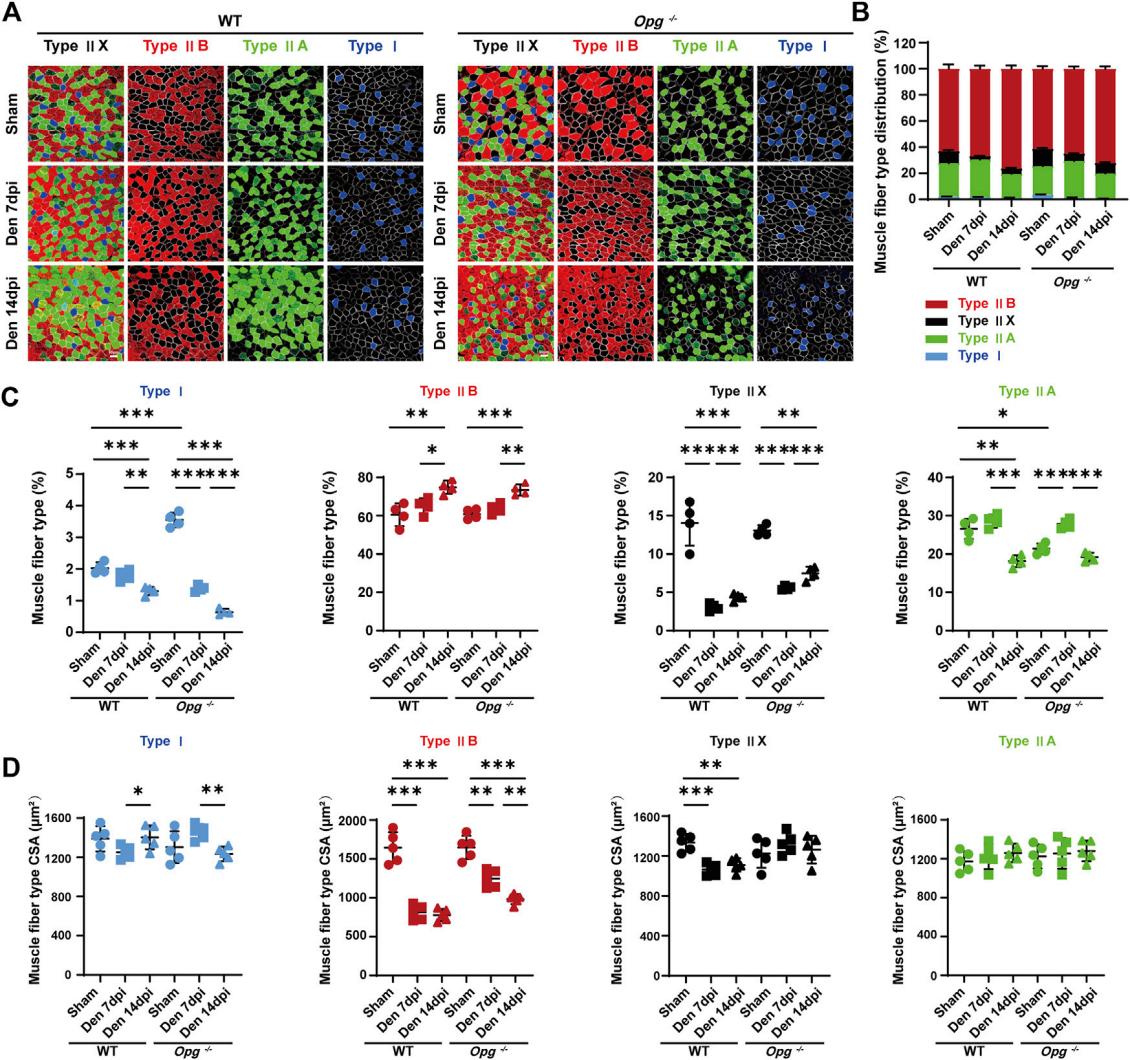
Denervation promoted muscle fiber type transition and *Opg* knockout delayed type ⅡB myofiber atrophy. **(A, B)** Representative immunofluorescence images and stacked bar graphs showing muscle fiber type composition and transition of gastrocnemius muscles days 7 and 14 after denervation relative to sham group. Scale bar 50 μm. **(C)** The percentage of gastrocnemius muscle fiber subtypes I (blue), IIB (red), IIX (black) and type IIA (green). **(D)** The mean CSA of gastrocnemius muscle fiber subtypes I (blue), IIB (red), IIX (black) and type IIA (green). *n* = 4-5/group. Data are represented as the mean ± SD. ∗*p* < 0.05, ∗∗*p* < 0.01, ∗∗∗*p* < 0.001.

To further explore the effects of *Opg* knockout on specific muscle fiber type, we performed muscle fiber phenotyping of the GAS muscle types I, IIA, IIB and IIX, the percentage of which could change due to physical exercise, disuse, aging and injury (You et al., 2021) (Figures 4A, B). In summary, denervation promoted a muscle fiber type switch from slow-to fast-twitch, reflecting the change in the metabolic states of myofibers. This was consistent for both denervated WT and *Opg* ^*−/−*^ mice (Figures 4A, B). Type I fibers (slow-twitch myofibers) increased in *Opg* ^*−/−*^ mice compared to WT mice (Figure 4C). Denervation induced the loss of type I fibers (%) in both WT and *Opg* ^*−/−*^ mice but was especially significant in *Opg* ^*−/−*^ mice (Figure 4C), whereas the CSA of type I fibers showed no significant difference (Figure 4D). In GAS muscles, type IIB fibers were the primary fiber type making up more than 60% of the total myofibers and type IIB fibers increased following denervation in both WT and *Opg* ^*−/−*^ mice (Figure 4C). Interestingly, the CSA of WT mice was significantly reduced upon denervation, whereas *Opg* knockout delayed the loss of CSA of type IIB fibers (Figure 4D). The percentage of other non-type IIB fibers fluctuated in both WT and *Opg* ^*−/−*^ mice, which was likely to be influenced by hybrid muscle fibers, such as types IIB/IIX, IIA/IIX and IIA/IIB fibers, but the percentage of other non-type IIB fibers decreased (Figure 4C). Moreover, the change in the CSA of other non-type IIB fibers was not significant, except for type IIX fibers in WT mice, which appeared as smaller fibers (Figure 4D). Taken together, denervation promoted muscle fiber type from slow-fast transformation and *Opg* knockout delayed denervated muscle atrophy in a specific type IIB fiber.

### 3.5 *Opg* knockout upregulated TET2 expression along with other muscle protective genes

**FIGURE 5:**
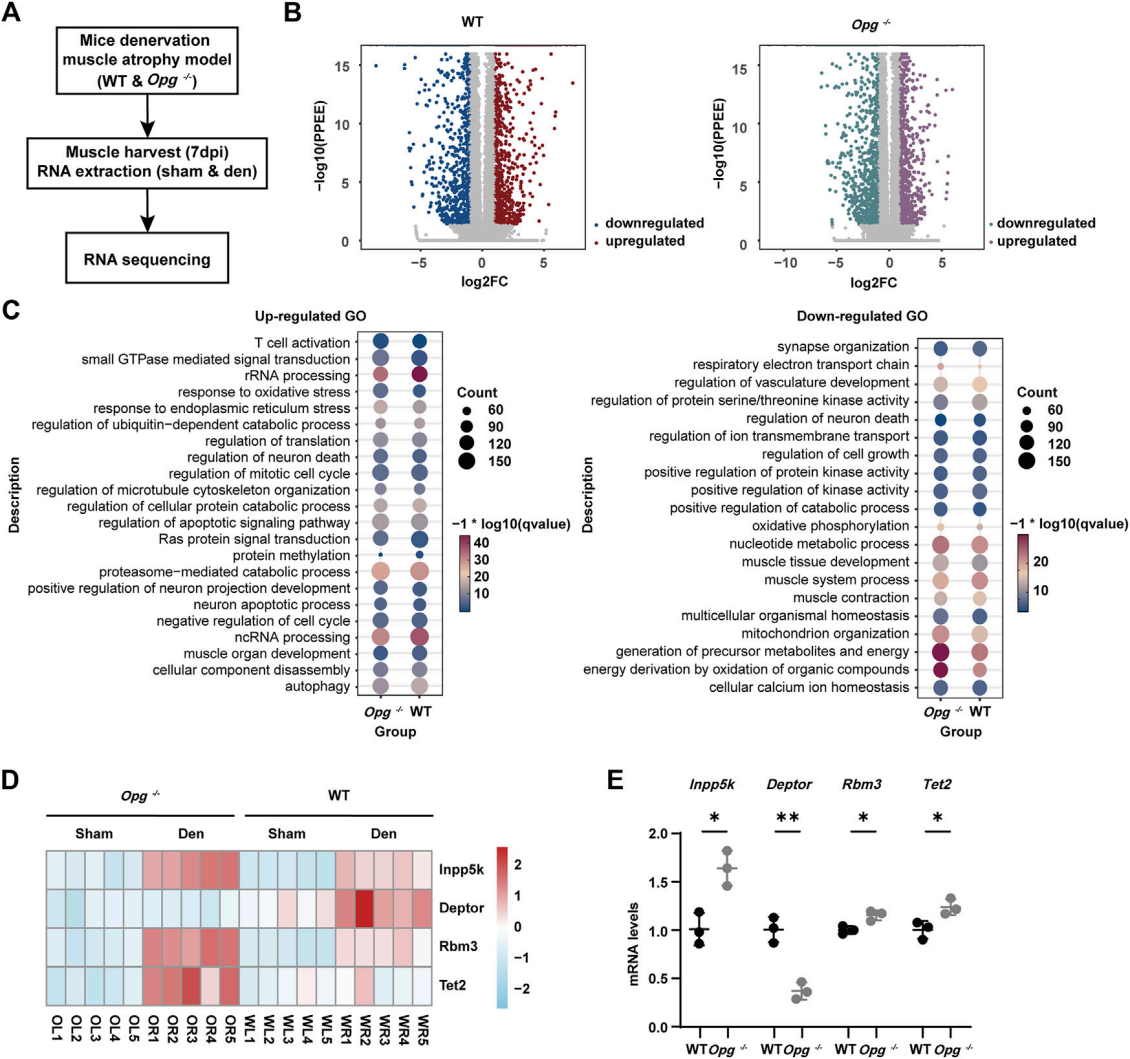
Effect of *Opg* knockout on the transcriptome of denervated GAS muscle. **(A)** Schematic of the experimental procedure for transcriptome sequencing and the identification of the differentially expressed genes in denervated muscle due to *Opg* knockout. **(B)** Volcano plot visualizing upregulated or downregulated genes after denervation in WT mice and *Opg* ^*−/−*^ mice. **(C)** Gene ontology analysis of upregulated or downregulated genes after denervation in WT and *Opg* ^*−/−*^ mice. **(D)** Heatmap showing the selected differentially expressed genes of WT and *Opg* ^*−/−*^ mice after denervation. **(E)** The mRNA expression of *Inpp5k*, *Deptor*, *Rbm3* and *Tet2* determined using qRT-PCR in gastrocnemius muscles of WT and *Opg* ^*−/−*^ mice 7 days after denervation. *n* = 5/group. Data are represented as the mean ± SD. ∗*p* < 0.05, ∗∗*p* < 0.01.

To further understand the underlying molecular mechanism of how *Opg* knockout delayed denervated muscle atrophy, we performed transcriptome sequencing of GAS muscles 7 days post denervation, the peak of phenotypic differences between WT and *Opg* ^*−/−*^ mice (Figure 5A). PCA and Pearson correlation analysis revealed clear sample clusters and correlations (Supplementary Figure S3). In WT mice, compared to the sham group, the expression of 3,028 genes was significantly changed in den group in which 1,495 were upregulated and 1,533 were downregulated (Figure 5B). In *Opg* ^*−/−*^ mice, compared to the sham group, the expression of 3,126 genes was significantly altered in den group in which 1,551 genes were upregulated and 1,575 genes were downregulated (Figure 5B). We also performed GO analysis of DEGs after denervation in WT and *Opg* ^*−/−*^ mice. Upregulated pathways were mainly involved in rRNA processing, endoplasmic reticulum stress, ubiquitin-dependent catabolic process, apoptotic signaling pathway, neuron death, muscle organ development and autophagy in both WT and *Opg* ^*−/−*^ mice (Figure 5C). Downregulated pathways involved synapse organization, vasculature development, oxidative phosphorylation, muscle system process and mitochondrion organization in both WT and *Opg* ^*−/−*^ mice (Figure 5C). There was no significant difference in terms of the GO terms. However, we did find some genes associated with muscle function differentially expressed between WT den group and *Opg* ^*−/−*^ den group, *Inpp5k*, *Deptor*, *Rbm3* and *Tet2*, and performed further validation using qRT-PCR (Figures 5D, E). We found that the expression of *Inpp5k* and *Rbm3* increased upon denervation in both WT and *Opg* ^*−/−*^ mice; however, *Opg* ^*−/−*^ mice showed higher expression levels (Figure 5D), which exerted protective effects against muscle atrophy (Hettinger et al., 2021; McGrath et al., 2021). In addition, *Deptor* expression was only elevated in WT mice (Figure 5D), knockdown of which could ameliorate disused muscle atrophy (Kazi et al., 2011). More importantly, the expression of *Tet2,* coding for an important DNA demethylase (Ostriker et al., 2021; Wang et al., 2021; Zhang et al., 2022), only increased in denervated *Opg* ^*−/−*^ mice (Figure 5D) Therefore, we speculated that TET2 might regulate the key transcription factors to further exert protective effects on muscles.

### 3.6 *Opg* knockout upregulated TET2 expression in differentiated SCs

**FIGURE 6:**
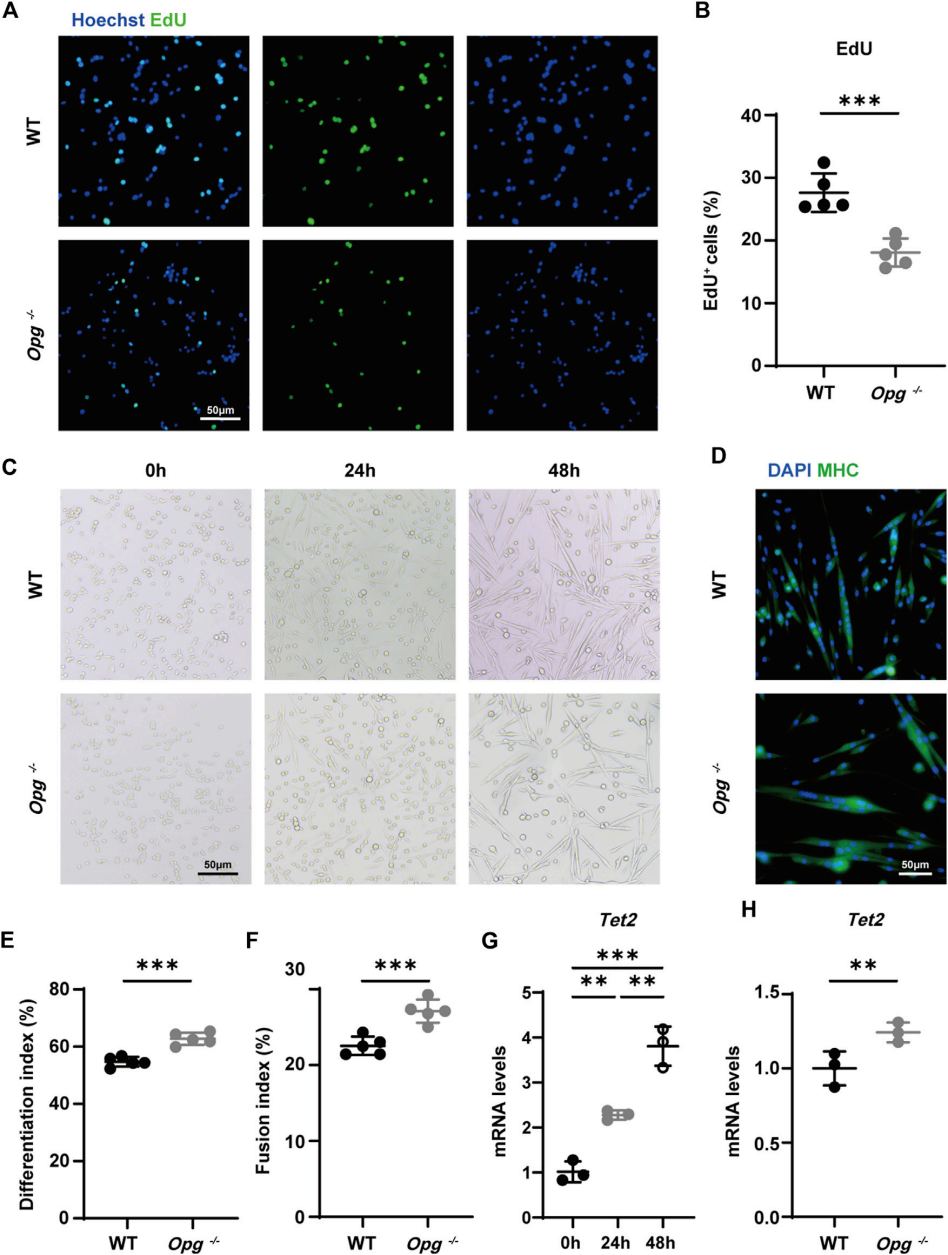
*Opg* knockout upregulated TET2 expression in differentiated SCs. **(A, B)** Representative immunofluorescence images **(A)** and quantification **(B)** of EdU to evaluate the proliferation ability of SCs obtained from WT and *Opg* ^*−/−*^ mice. Scale bar 50 μm. **(C)** Representative bright field images of WT and *Opg* ^*−/−*^ SCs differentiation at indicated time points. Scale bar 50 μm. **(D)** Representative immunofluorescence images of MHC-stained myotubes 48 h after the induction of differentiation. Scale bar 50 μm. **(E, F)** Differentiation index **(E)** and fusion index **(F)** to evaluate the differentiation ability of SCs of the indicated groups. **(G)** The mRNA expression of *Tet2* determined using qRT-PCR in differentiated WT SCs. **(H)** The mRNA expression of *Tet2* determined using qRT-PCR for the indicated groups 48 h after induction of differentiation. *n* = 3–5/group. Data are represented as the mean ± SD. ∗∗*p* < 0.01, ∗∗∗*p* < 0.001.

We sorted primary SCs from WT and *Opg* ^*−/−*^ mice *via* FACS to explore the effects of *Opg* knockout. According to previous report, we harvested hindlimb muscles from 6-week-old male WT and *Opg* ^*−/−*^ mice and obtained α7-integrin^+^, 7-AAD^-^ and Lin^−^ (CD11b/CD31/CD45/Sca1) SCs (Supplementary Figure S4) (Ryall et al., 2015). First, we evaluated the proliferation ability of SCs from WT and *Opg* ^*−/−*^ mice using EdU staining and found that the number of EdU^+^ cells in WT mice was significantly higher than that in *Opg* ^*−/−*^ mice, suggesting that *Opg* knockout inhibited cell proliferation (Figures 6A, B), which was reported that OPG was a potent mitogen (Arnold et al., 2019). Moreover, we evaluated the differentiation ability of SCs *via* myotube staining and found that *Opg* knockout SCs showed better differentiation ability, as evidenced by a higher differentiation index and fusion index compared to WT SCs (Figures 6C–F). Lastly, we verified the expression of *Tet2* during SCs differentiation and found the increasing expression of *Tet2* in SCs differentiation and *Opg* ^*−/−*^ SCs had higher *Tet2* expression than WT ones (Figures 6G, H). In general, OPG deficiency inhibited SC proliferation and promoted SC differentiation accompanied by higher *Tet2* expression.

## 4 Discussion

The role of the OPG/RANKL/RANK axis in bone metabolism has been well elucidated, but its function in muscles has not been fully characterized. Previous studies have preliminarily explored the effects of the OPG/RANKL/RANK pathway on muscle and suggest that *Opg* knockout mice with high RANKL level exhibit selective muscle atrophy, and anti-RNAKL, RANK deletion and OPG-Fc treatments alleviate muscle atrophy in *mdx* dystrophic mice (Dufresne et al., 2018; Hamoudi et al., 2020). Therefore, we infer that OPG deficiency and high RANKL expression may aggravate muscle atrophy and harm muscle function in denervated *Opg* knockout mice. Intriguingly, our results show the opposite tendency that *Opg* knockout delays denervated muscle atrophy and decreases muscle fibrosis, as evidenced by the higher wet weight ratio and lower fibrotic area in *Opg* knockout mice compared to WT mice. Regardless of denervation, *Opg* knockout mice exhibit undergoing muscle atrophy and lower CSA of GAS than WT mice, whereas the CSA of *Opg* knockout mice is comparable with WT mice after denervation (Figure 2E) indicating that *Opg* knockout indeed exerts potential protective effects on denervated muscle atrophy.

The different outcomes of the OPG/RANKL/RANK axis in animal models may reflect the different role of this axis under various physiological and pathological conditions. For example, the selective inhibition of RANKL in hematopoietic cells improves myocardial infarction outcome (Slavic et al., 2018). Conversely, increased RANKL/RANK signaling in *Opg* ^*−/−*^ mice and recombinant RANKL treatment reduce the ischemic brain infarct volume and brain edema (Shimamura et al., 2014). Moreover, RANKL treatment before ischemia or at reperfusion protects against hepatic ischemia/reperfusion injury *via* hepatocyte NF-κB activation (Sakai et al., 2012). And RANKL partial peptide protects against bleomycin-induced pulmonary fibrosis (Ju et al., 2022). Consequently, it is reasonable that the role of the OPG/RANKL/RANK axis may depend on muscle research models. Previous studies mainly focus on muscle development, muscle injury and inherited muscular atrophy, such as cardiotoxin-induced muscle injury and *mdx* muscular dystrophy model, which rely on muscle growth, muscle injury and regeneration mechanisms (Bouredji et al., 2021; Hamoudi et al., 2020). However, few studies focus on the role of the OPG/RANKL/RANK axis in secondary muscle atrophy by different stimulus, such as glucocorticoid, cancer cachexia, malnutrition, denervation, immobilization and hindlimb suspension. Studies show that despite of the same muscle atrophy outcome, different molecular mechanisms and largely different mRNA and protein changes are associated with distinct modes of skeletal muscle atrophy (Bonaldo and Sandri, 2013; Hunt et al., 2021). For example, PI3K/AKT/mTOR pathway is important for muscle protein synthesis. mTOR activation alleviates glucocorticoid-induced muscle atrophy and prevents muscle atrophy during immobilization, whereas denervation-induced atrophy is not dependent upon the activation of AKT and mTOR and is not rescued by myostatin inhibition (Bonaldo and Sandri, 2013; MacDonald et al., 2014; You et al., 2015). Also, studies show that the activation of mTOR signaling during denervation increases catabolic signaling events, but activation of mTOR signaling during immobilization exerts anabolic effect on protein synthesis (You et al., 2015). Moreover, ceramide accumulation and lipid raft disruption after hindlimb suspension are atrophy-promoting early events, whereas denervation can increase lipid raft formation and integrity (Bryndina et al., 2021). Furthermore, an important protein degradation mechanism, autophagy decreases in aging, *mdx* muscular dystrophy and collagen VI-null mice model, whereas autophagy is activated in denervation and immobilization (Chrisam et al., 2015; Yang et al., 2018). In general, different stimulus induce muscle atrophy *via* different mechanisms and denervated muscle atrophy is involved in increased protein degradation, increased oxidative stress, activated autophagy, mitochondrial dysfunction, lasting inflammation and fibrosis (Rebolledo et al., 2019), which complement the function of the OPG/RANKL/RANK axis in muscles from a different perspective by modeling denervation-induced muscle atrophy. Similarly, Dufresne *et al.* show that RANK deletion promotes atrophy in denervated extensor digitorum longus muscles (Dufresne et al., 2016), indicating that RANKL inactivity exacerbates muscle atrophy during denervation. In our experiment, OPG deficiency and high RANKL expression delayed denervated muscle atrophy.

Our transcriptome sequencing analysis suggested that denervation mainly increased endoplasmic reticulum stress, ubiquitin-dependent catabolic processes and autophagy and significantly decreased vasculature development and mitochondrial organization. We found some interesting DEGs in the comparison between denervated GAS in *Opg* ^*−/−*^ mice and WT mice, such as *Inpp5k*, *Deptor*, *Rbm3* and *Tet2*. Previous reports suggest that suppressed autophagy causes skeletal muscle disease in *Inpp5k* ^*−/−*^ mice and *Deptor* knockdown ameliorates disused muscle atrophy (Kazi et al., 2011; McGrath et al., 2021). RBM3 exerts protective effects on skeletal muscle, attenuates atrophy and induces hypertrophy (Ferry et al., 2011; Hettinger et al., 2021; Van Pelt et al., 2018). More importantly, TET2 is an important DNA demethylase engaging in various biological processes (Wang et al., 2021). For example, TET2 has been proven to protect against smooth muscle cell apoptosis and mediate smooth cell differentiation (Ostriker et al., 2021; Zhang et al., 2022). TET2 loss impairs muscle morphology and causes severe regeneration defects. TET2 is also necessary for the proliferation and differentiation of muscle stem cells (Wang et al., 2021; Zhang et al., 2022). Other studies have shown increased expression of TET2 during RANKL-induced osteoclast differentiation (Yang et al., 2022). In our study, we reported higher expression levels of *Tet2* in *Opg* ^*−/−*^ mice after denervation and differentiated SC cells from *Opg* ^*−/−*^ mice. Therefore, we speculate that TET2 induces epigenetic modification of transcription factors, which may exert a protective effect in denervated muscles. Details of the interaction between *Opg* knockout, TET2 and target genes have not yet been directly investigated but will be the focus of future research.

In addition to delayed muscle atrophy, we also showed that *Opg* knockout decreased muscle fibrosis during denervation, an important muscle degeneration phenotype which usually emerge in aging people. Though lacking in-depth exploration in our study, Wang *et al.* confirm that RANKL binds to RANK on macrophages and initiates the extracellular matrix degradation, whereas increased OPG levels in fibrotic tissues inhibit this process (Wang et al., 2019). In this manuscript, our results reveal the impact of the OPG/RANKL/RANK pathway dysfunction on denervated muscle atrophy and indicate that it may be a promising novel target of muscle degeneration. Moreover, the effects of *Opg* knockout and high RANKL on muscle highlight the potential side effects or common therapeutic effects of RANKL inhibitors in clinical use, such as the association between muscle fibrosis and RANKL inhibitors. Furthermore, the systematic effects of RANKL inhibitors are also worthy of comprehensive research, especially in the most common degenerative organs of older people, such as the lungs, liver and heart. In summary, this study explored the novel role of OPG/RANKL/RANK pathway in muscle and revealed that *Opg* knockout delayed denervated muscle atrophy and upregulated *Tet2* expression in GAS muscles.

## Data Availability

The datasets presented in this study can be found in online repositories. The names of the repository/repositories and accession number (s) can be found in the article/Supplementary Material.
