# Supplementary material for: Delayed denervation-induced muscle atrophy in Opg knockout mice
Source: Front Physiol. 2023 Feb 22;14:1127474. doi: 10.3389/fphys.2023.1127474 (PMC9992212; doi:10.3389/fphys.2023.1127474)
Supplement: Supplementary file 1 [file DataSheet2.PDF]

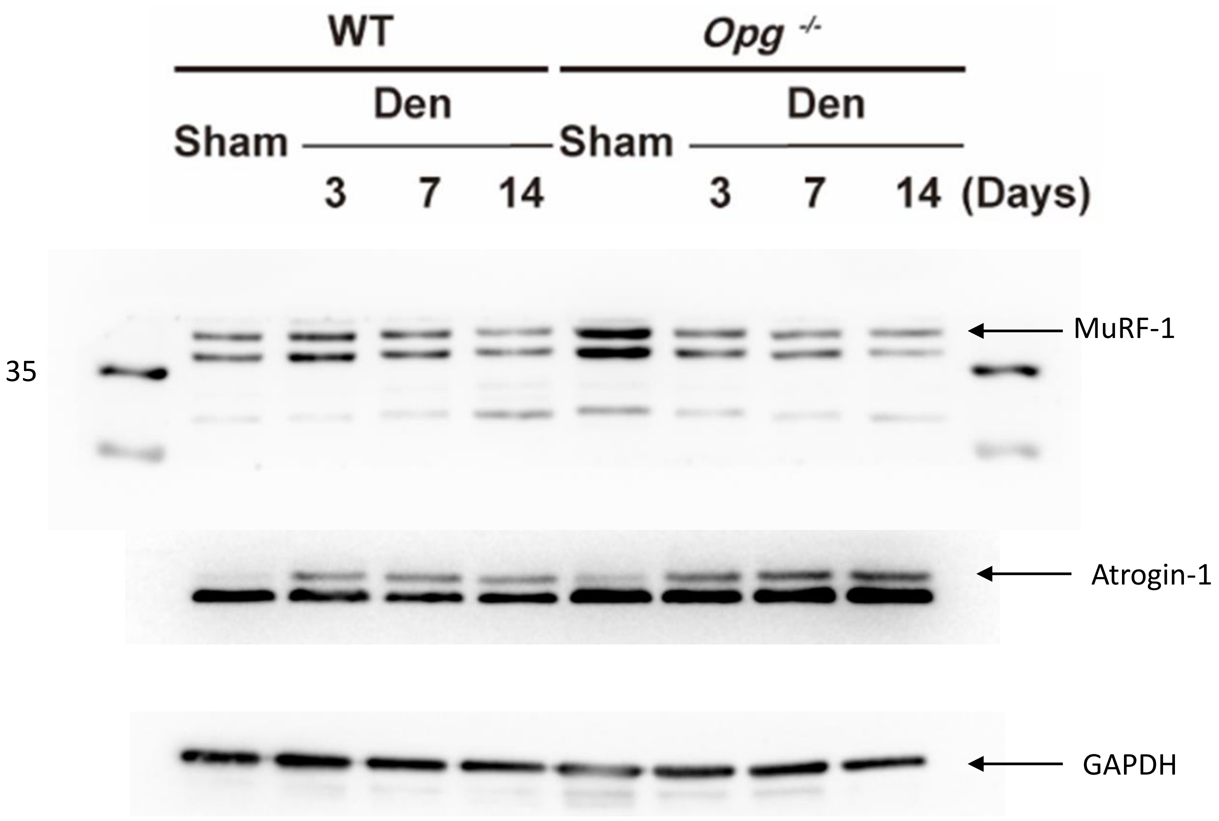
WT
Opg -/-
Sham
Den
3
7
14
Sham
Den
3
7
14 (Days)
35
MuRF-1
Atrogin-1
GAPDH

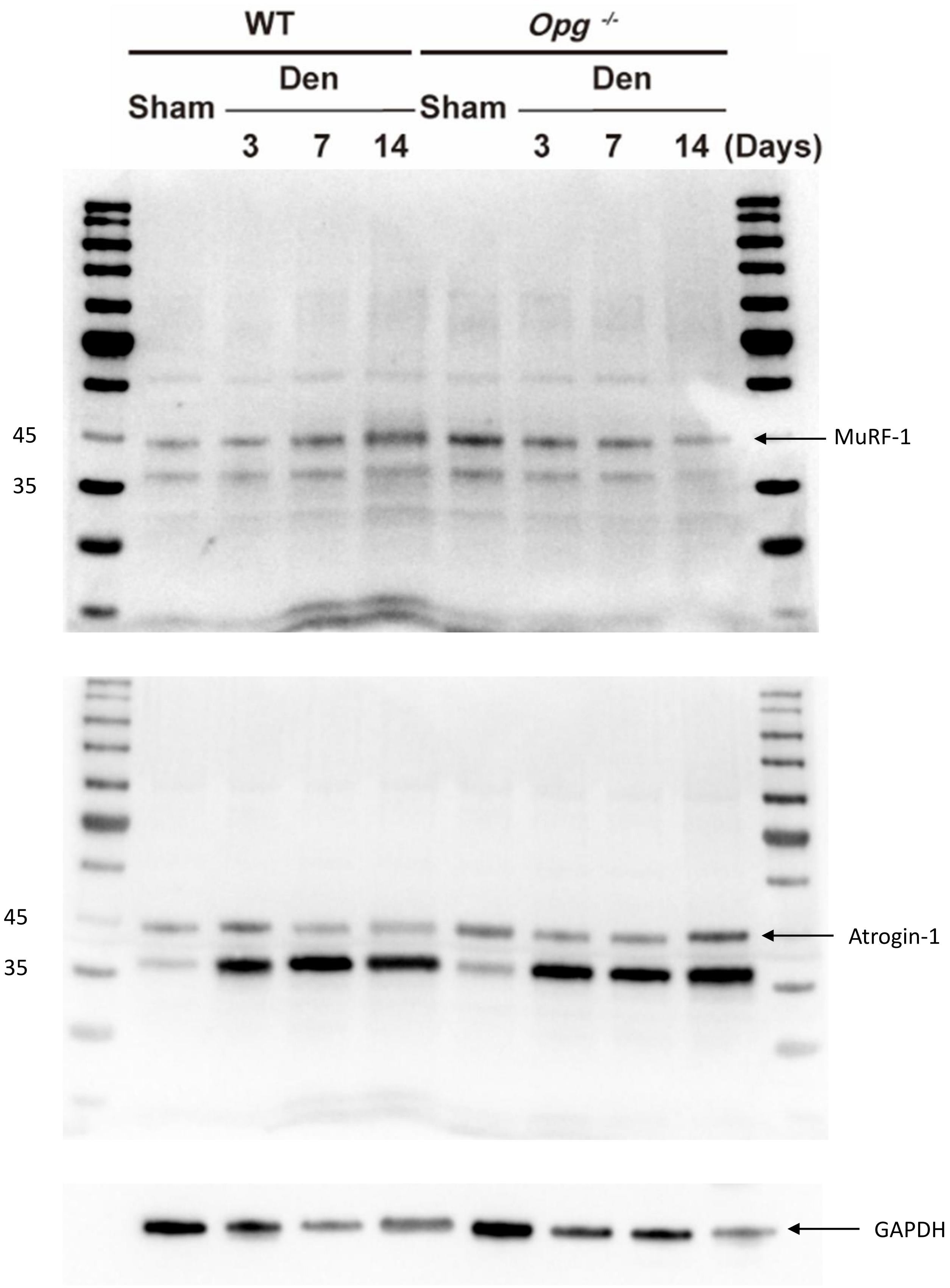
WT
Opg -/-
Sham
Den
3
7
14
Sham
Den
3
7
14 (Days)
45
35
MuRF-1
45
35
Atrogin-1
GAPDH

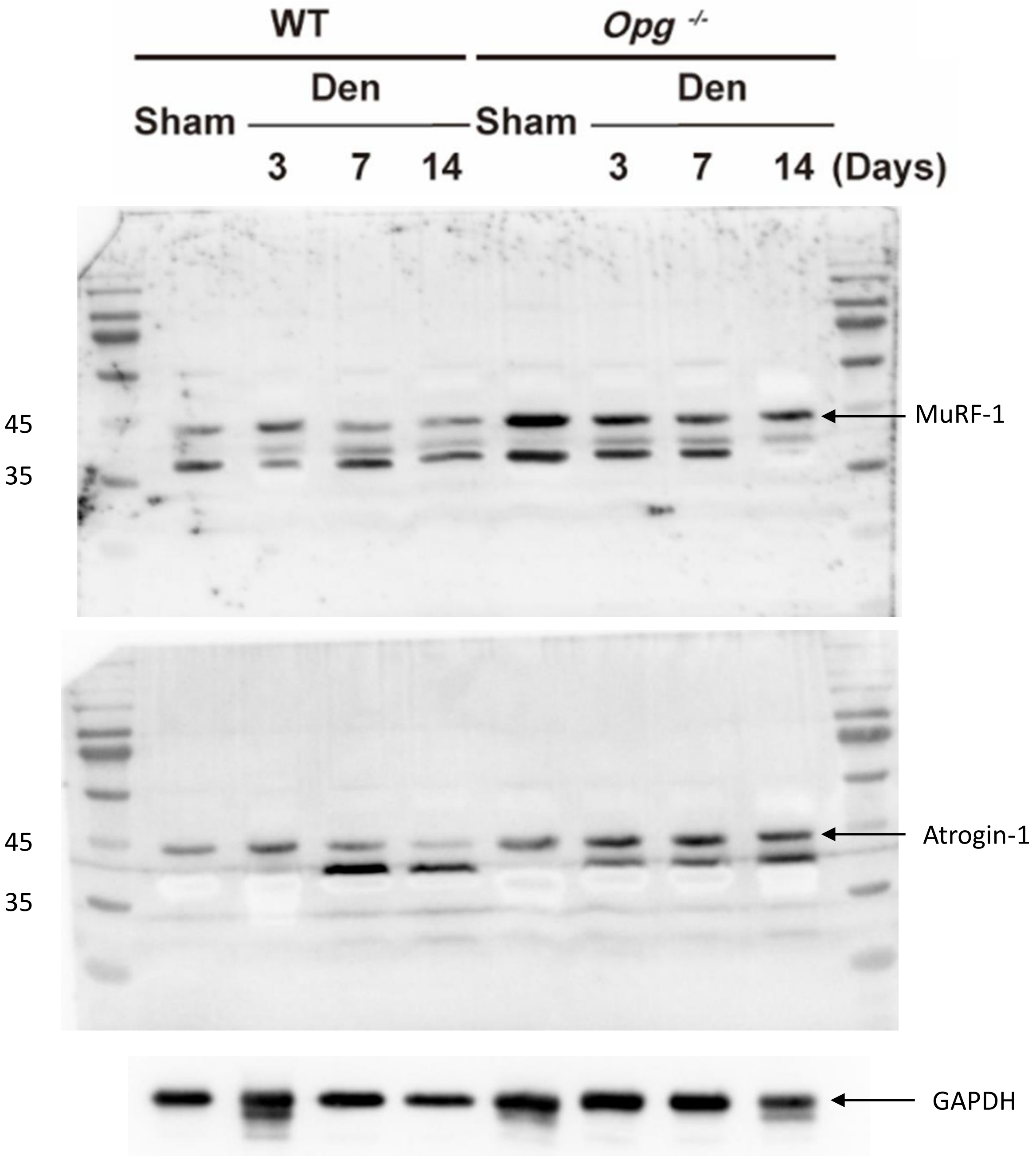

WT
Opg -/-
Sham
Den
3
7
14
Sham
Den
3
7
14
(Days)
45
35
MuRF-1
45
35
Atrogin-1
GAPDH
