# Supplementary material for: Delayed denervation-induced muscle atrophy in Opg knockout mice
Source: Front Physiol. 2023 Feb 22;14:1127474. doi: 10.3389/fphys.2023.1127474 (PMC9992212; doi:10.3389/fphys.2023.1127474)
Supplement: Supplementary file 2 [file DataSheet1.DOCX]

Supplementary Material

Delayed denervation-induced muscle atrophy in *Opg* knockout mice

**Mingming Zhang^†^, Ming Chen^†^, Yi Li, Man Rao, Duanyang Wang, Zhongqi Wang, Licheng Zhang**^*^**, Pengbin Yin**^*^**, Peifu Tang**

^†^These authors contributed equally to this work.

*** Correspondence:**

Licheng Zhang

[zhanglcheng218@126.com](mailto:zhanglcheng218@126.com)

Pengbin Yin

yinpengbin@gmail.com

# Supplementary Figures


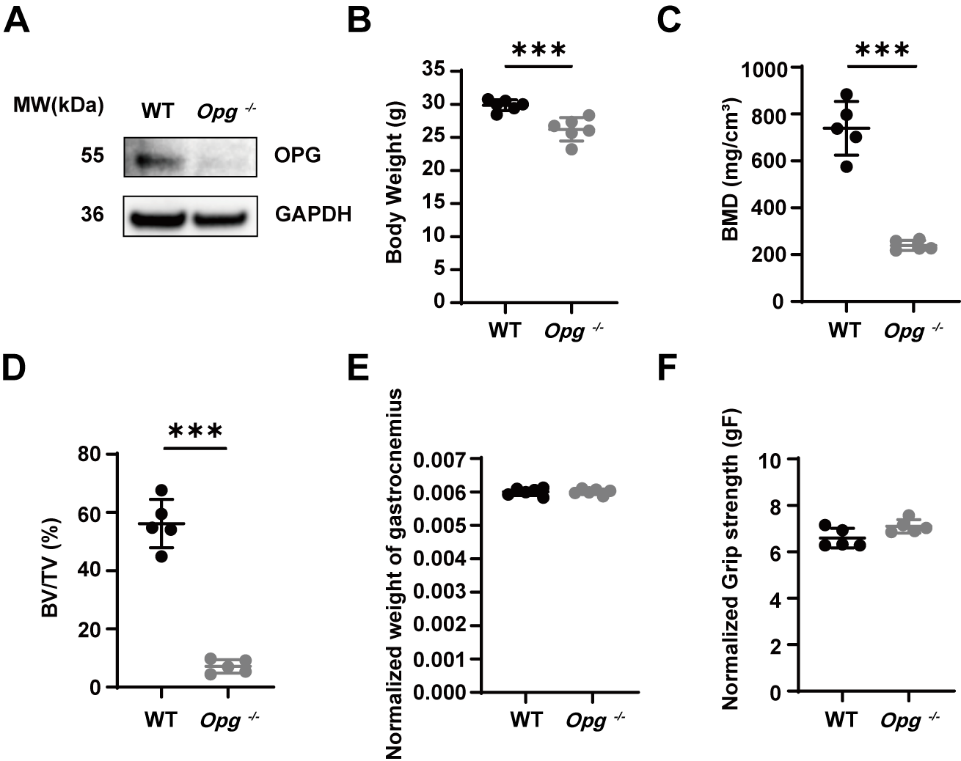


**Supplementary Figure 1 Characterization of the phenotypes of *Opg^-/-^* mice.** (A) Western blotting results showing OPG expression in WT and *Opg^-/-^* mice. (B) Comparison of the body weight of 12-week-old WT and *Opg^-/-^* mice. (C, D) Micro-CT evaluation of femurs of 12-week-old WT and *Opg^-/-^* mice, including BMD (C) and BV/TV (D). (E) Quantification of the GAS muscle weight of 12-week-old WT and *Opg^-/-^* mice normalized to body weight. (F) Grip strength of 12-week-old WT and *Opg^-/-^* mice normalized to body weight. n = 5/group. Data are represented as the mean ± SD. ∗∗∗*P* < 0.001.

**
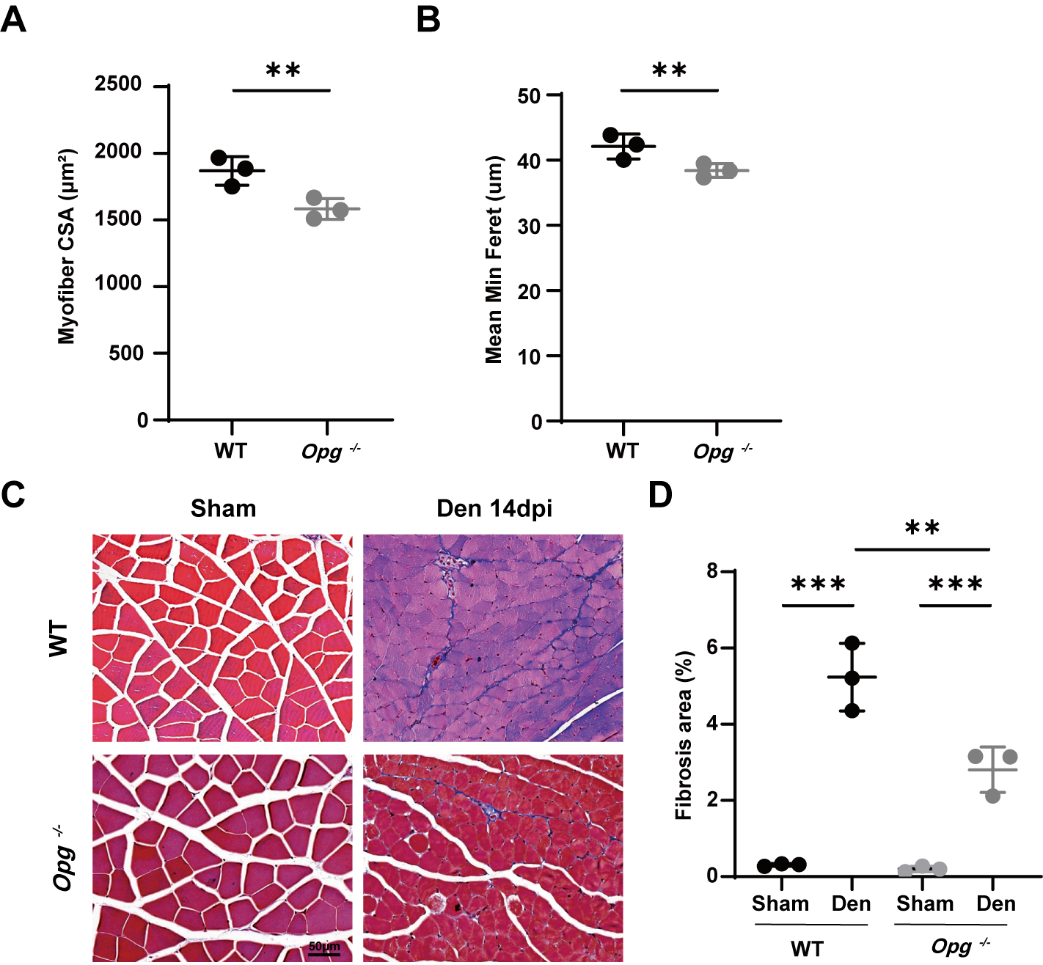
**

**Supplementary Figure 2 *Opg* knockout induced muscle atrophy and *Opg* knockout alleviated muscle fibrosis after denervation.** (A, B) Myofiber CSA (A) and mean minimal Feret diameter (B) of GAS muscles of 12-week-old WT and *Opg^-/-^* mice. (C, D) Representative Masson’s trichrome staining images (C) and quantification of fibrotic area (D) of GAS muscles 14 days after denervation compared to sham group. Scale bar 50 μm. n = 3/group. Data are represented as the mean ± SD. ∗∗*P* < 0.01, ∗∗∗*P* < 0.001.


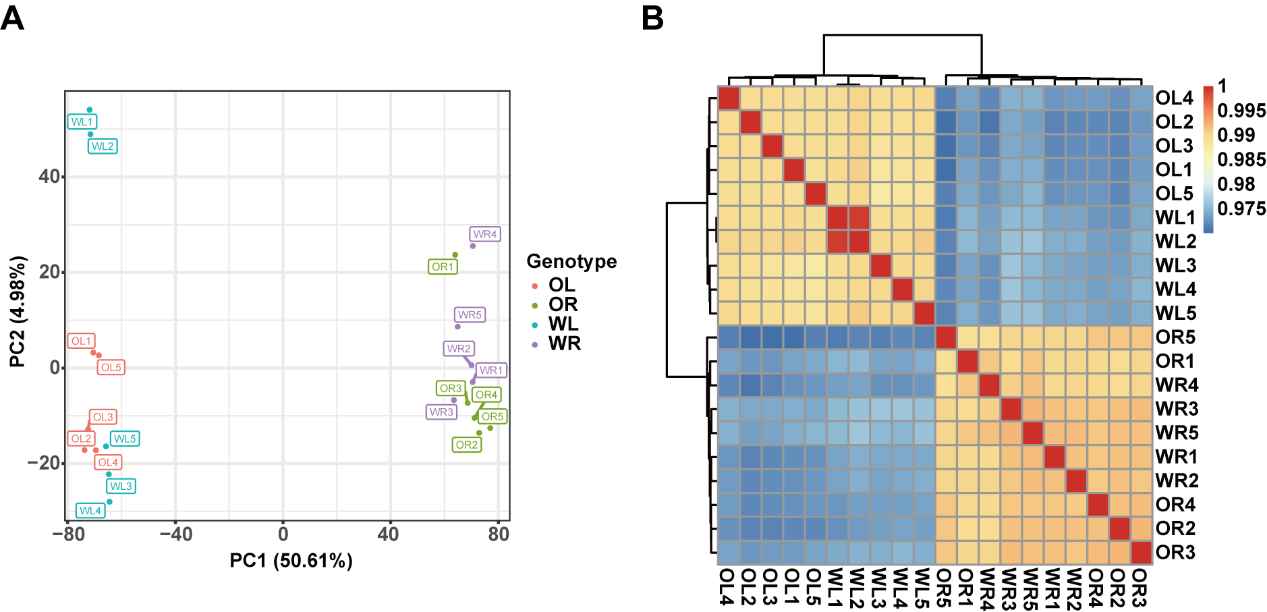


**Supplementary Figure 3 Clustering and correlation analysis of the sequencing samples.** (A, B) Principal component analysis (PCA) (A) and Pearson correlation analysis (B) of den and sham samples of WT and *Opg^-/-^* mice.


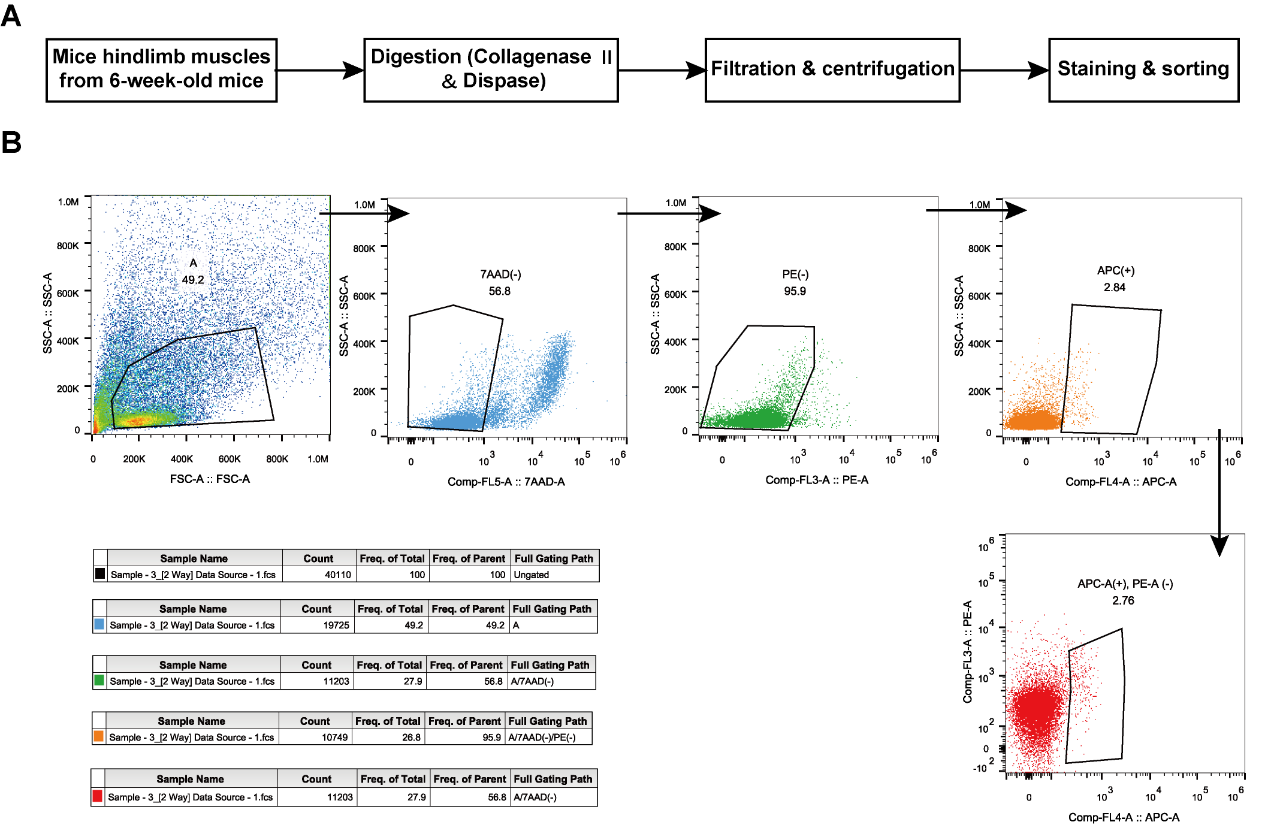


**Supplementary Figure 4 SCs were isolated using FACS.** (A) Schematic of the digestion and staining procedure used for the isolation of SCs using FACS. (B) Representative FACS scatter plots visualizing sorted SCs.

# Supplementary Information for Materials and Methods

**Supplementary Table 1** Antibodies used

| **Product name** | **Source and**  **Catalog #** | **Host species** | **Application** | **Dilution** |
| --- | --- | --- | --- | --- |
| Anti-MuRF-1 | Abcam, ab172479 | Rabbit | WB | 1:1000 |
| Anti-Fbx32 | Abcam, ab168372 | Rabbit | WB | 1:1000 |
| Anti-OPG | Santa Cruz,  sc-390518 | Mouse | WB | 1:100 |
| Anti-GAPDH | Abcam, ab8245 | Mouse | WB | 1:5000 |
| Anti-mouse IgG H&L (HRP) | Abcam, ab205719 | Goat | WB | 1:5000 |
| Anti-rabbit IgG H&L (HRP) | Abcam, ab6721 | Goat | WB | 1:10000 |
| Anti-Myhc Ⅰ | DSHB, BA-D5 | Mouse | IF | 1:6 |
| Anti-Myhc IIa | DSHB, SC-71 | Mouse | IF | 1:10 |
| Anti-Myhc IIb | DSHB, BF-F3 | Mouse | IF | 1:10 |
| Anti-Dystrophin | Abcam, ab15277 | Rabbit | IF | 1:200 |
| Anti-rabbit IgG Alexa Fluor® 405 | Abcam, ab175652 | Goat | IF | 1:500 |
| Anti-mouse IgM Alexa Fluor® 488 | Abcam, ab150121 | Goat | IF | 1:2000 |
| Anti-Mouse IgG1 Alexa Fluor™ 568 | Invitrogen, A-21124 | Goat | IF | 1:500 |
| Anti-mouse IgG2b Alexa Fluor® 647 | Abcam, ab172327 | Rat | IF | 1:200 |
| APC-labeled α7-integrin | Invitrogen,  MA5-23555 | Rat | F | 1:200 |
| PE-labeled CD11b | BD, 557397 | NA | F | 1:200 |
| PE-labeled CD31 | BD, 553373 | NA | F | 1:200 |
| PE-labeled CD45 | BD, 553081 | NA | F | 1:200 |
| PE-labeled Sca1 | BD, 553108 | NA | F | 1:200 |
| Anti-MHC | DSHB, MF20 | Mouse | ICC | 4μg/ml |
| Anti-mouse  Alexa Fluor® 568 | Abcam, ab175473 | Goat | ICC | 1:500 |

**Supplementary Table2** Reagents used

| **Product name** | **Source** | **Catalog #** |
| --- | --- | --- |
| Hematoxylin-Eosin (HE) stain kit | Solarbio | G1120 |
| Modified Masson’s Trichrome stain kit | Solarbio | G1346 |
| QuickBlock™ blocking buffer | Beyotime | P0260 |
| QuickBlock™ primary antibody dilution buffer | Beyotime | P0262 |
| QuickBlock™ secondary antibody dilution buffer | Beyotime | P0265 |
| Protease inhibitor cocktail | Roche | 4693116001 |
| BCA protein concentration determination kit | Beyotime | P0010 |
| EveryBlot blocking buffer | Bio-Rad | 12010020 |
| Clarity Western ECL Substrate | Bio-Rad | 170-5061 |
| FastPure® Cell/Tissue Total RNA Isolation Kit V2 | Vazyme Biotech | RC112-01 |
| HiScript® Ⅲ All-in-one RT SuperMix Perfect for qPCR | Vazyme Biotech | R333-01 |
| ChamQ Universal SYBR qPCR Master Mix | Vazyme Biotech | Q711-02/03 |
| TRIzol | Beyotime | R0016 |
| Collagenase II | Sigma-Aldrich | C6885 |
| Dispase | Sigma-Aldrich | D4693 |
| Collagen Ⅰ | Solarbio | C8062 |
| Basic fibroblast growth factor | Promega | G5071 |
| Dulbecco's Modified Eagle Medium | Gibco | 10569010 |
| BeyoClickTM EdU cell proliferation kit | Beyotime | C0075S |

**Supplementary Table 3** Primers used

|  | **Forward** | **Reverse** |
| --- | --- | --- |
| Inpp5k | CAGCACGGAGACAGGAACAC | AGGCCACATTCCACGTCAC |
| Deptor | ATAGACGGCACCATCTCAAAAC | GTCGGCTAATTTCTGCATGAGT |
| Rbm3 | CTTCGTAGGAGGGCTCAACTT | CTCCCGGTCCTTGACAACAAC |
| Tet2 | GTGCTGCTGGATTCATTCAAAG | GTATGGCTGATGGGAGAAGGTG |
| Gapdh | CAACTCCCTCAAGATTGTCAGCAA | GGCATGGACTGTGGTCATGA |
